# Self-Assembled Hydrogel Membranes with Structurally Tunable Mechanical and Biological Properties

**DOI:** 10.1021/acs.biomac.4c00082

**Published:** 2024-05-13

**Authors:** Rasha M. Abdel-Rahman, A. M. Abdel-Mohsen, Jana Frankova, Francesco Piana, Lukas Kalina, Veronika Gajdosova, Ludmila Kapralkova, Muhammed Arshad Thottappali, Josef Jancar

**Affiliations:** † CEITEC-Central European Institute of Technology, Brno University of Technology, Purkyňova 656/123, Brno 61200, Czech Republic; ‡ Institute of Macromolecular Chemistry, Czech Academy of Sciences, Heyrovského nám. 2, Praha 162 06, Czech Republic; § Pretreatment and Finishing of Cellulosic Based Textiles Department, Textile Industries Research Institute, National Research Centre, 33 EL Buhouth Street, Dokki, Giza 12622, Egypt; ∥ Department of Medical Chemistry and Biochemistry, Faculty of Medicine and Dentistry, Palacký University, Hněvotínská, 3, 775 15, Olomouc, Czech Republic; ⊥ Faculty of Chemistry, Materials Research Centre, Brno University of Technology, Purkyňova 464/118, Brno 61200, Czech Republic

## Abstract

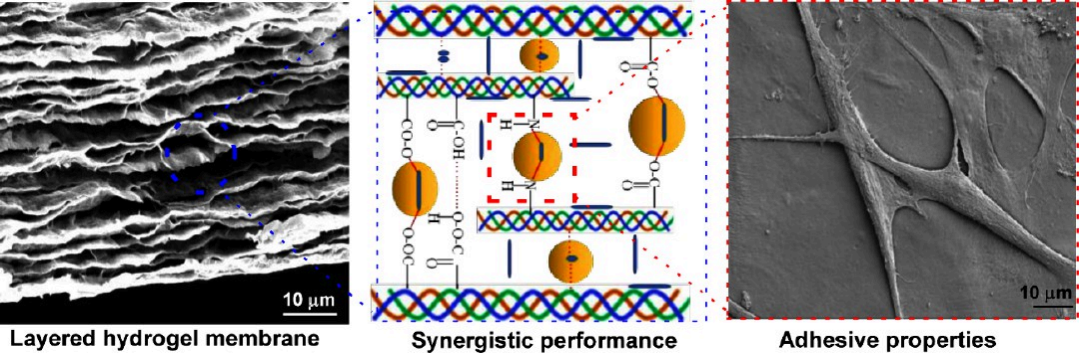

Using supramolecular self-assembled nanocomposite materials made from protein and polysaccharide components is becoming more popular because of their unique properties, such as biodegradability, hierarchical structures, and tunable multifunctionality. However, the fabrication of these materials in a reproducible way remains a challenge. This study presents a new evaporation-induced self-assembly method producing layered hydrogel membranes (LHMs) using tropocollagen grafted by partially deacetylated chitin nanocrystals (CO-*g*-ChNCs). ChNCs help stabilize tropocollagen’s helical conformation and fibrillar structure by forming a hierarchical microstructure through chemical and physical interactions. The LHMs show improved mechanical properties, cytocompatibility, and the ability to control drug release using octenidine dihydrochloride (OCT) as a drug model. Because of the high synergetic performance between CO and ChNCs, the modulus, strength, and toughness increased significantly compared to native CO. The biocompatibility of LHM was tested using the normal human dermal fibroblast (NHDF) and the human osteosarcoma cell line (Saos-2). Cytocompatibility and cell adhesion improved with the introduction of ChNCs. The extracted ChNCs are used as a reinforcing nanofiller to enhance the performance properties of tropocollagen hydrogel membranes and provide new insights into the design of novel LHMs that could be used for various medical applications, such as control of drug release in the skin and bone tissue regeneration.

## 1 Introduction

Nacre-like materials from natural biomaterials have potential applications in different fields, including tissue engineering, regenerative medicine, biomineralization studies, and biomaterial coatings.^1−5^ These materials can serve as scaffolds to promote cell adhesion, proliferation, and tissue regeneration in tissue engineering. They can also be used as substrates to study biomineralization processes and to form calcium carbonate-based structures. In addition, they can be applied as coatings on medical devices or implants to improve biocompatibility, reduce inflammation, and improve integration with surrounding tissues. Using the properties of natural biomaterials and mimicking the layered structure of nacre, these materials offer opportunities to develop advanced biomaterials with improved functionalities and performance for various biomedical applications.^4,6^

Collagen is the most abundant natural protein found in the extracellular matrix of different tissues and organs in mammals. It is commonly used in tissue engineering and regenerative medicine applications due to its biocompatibility and the ability to promote cell adhesion and proliferation.^5,7,8^ However, collagen-based hydrogels generally have limited mechanical strength and structural stability.^5^ Introducing nanoparticles into the collagen matrix allows the resulting nanocomposite hydrogel to exhibit improved mechanical properties, such as increased strength, modulus, and toughness. Nanoparticles can strengthen the collagen network, provide structural support, and prevent collapse or disintegration of the hydrogel structure.^9^ The choice of nanoparticles for the nanocomposite hydrogel depends on the desired properties and applications. Commonly used nanoparticles include inorganic materials such as silica,^10,11^ gold,^12,13^ silver^14−16^ and organic nanoparticles such as nanocellulose.^17^ These nanoparticles can be incorporated into the collagen matrix through various methods, including physical mixing,^8^ coassembly,^18^ or cross-linking.^13,19^

Chitin nanocrystals are promising nanofiller materials with high biocompatibility, biodegradability, excellent mechanical properties, and antibacterial activity.^20^ Chitin nanowhiskers have been used as nanofiller material to improve the mechanical properties of natural polymers such as hyaluronan,^21,22^ chitosan^23,24^ and synthetic polymers such as PMMA.^25^ The chitinous matrix promotes different bone cells (mesenchymal and osteoblast stem) and the ingrowth surrounding tissues.^26,27^ Chitin microfibers were used to prepare collagen/chitin composite scaffolds to improve the biological properties of collagen. However, this study does not show the effect of chitin on the mechanical and piezoelectric properties of collagen and the chemical interaction between collagen and chitin.^28^ Another study focused on the preparation of sponge-like collagen with polyvinylpyrrolidone (PVP) in the presence of chitin microparticles to improve the hemostasis properties of the wound dressing sheet.^29^ A hybrid scaffold from β-chitin and collagen was prepared using freeze-drying techniques from a chitin macromolecule solution dissolved in a lithium chloride/dimethylacetamide for the regeneration of bone tissue.^30^ The results show that the scaffold lacks mechanical properties after the chitin scaffold is treated with a different ratio of collagen solution.^30^ In addition, few studies focused on the preparation of collagen composite materials to improve their properties using chitin nanocrystals. Unfortunately, no data on mechanical properties, collagen denaturation during the preparation process, toxic solvents used, low biocompatibility and no synergetic properties between collagen and chitin were provided.^19,28,31^

Another common feature of these two biomaterials is that tropocollagen and chitin nanocrystals are self-assembled under controlled conditions (pH, temperature and concentration), leading to precisely assembled micro–and nanostructures. Synergistic effects of the combination of protein and polysaccharide during self-assembly in an aqueous solution have never been investigated.

This paper focuses on three main goals: (i) Development of layered flexible hydrogel membranes resembling the structure of nacre. Fabricating these materials will involve the combination of tropocollagen and partially deacetylated chitin nanocrystals employing evaporation-induced self-assembly avoiding the use of hazardous solvents. (ii) Investigation of the effects of ChNCs on mechanical physiological and piezoelectric properties of CO hydrogel membranes. (iii) Investigation of the relationships between chemical composition and biocompatibility of membranes using skin and bone cells.

## 2 Experimental Procedures

### 2.1 Materials and Methods

Acid insoluble collagen type I (CO) was purchased from VUP, Brno, Czech Republic, in the wet state with a dry mass of approximately 8 wt %. Native chitin from crab shells (Ch), 3-(4,5-dimethylthiazol-2-yl)-2,5-diphenyltetrazolium bromide, fetal bovine serum, Dulbecco’s modified Eagle medium, authenticated cell culture (ECACC), DMSO, ammonia solution, potassium chloride, sodium chloride and potassium dihydrogen phosphate were purchased from Sigma-Aldrich (Darmstadt, Germany). Hydrochloric acid, ethanol, and isopropyl alcohol were purchased from the Penta Chemical Company (Prague, Czech Republic). Octenidine dihydrochloride (OCT) was obtained from TCI Company (Sofia, Bulgaria). All reagents and chemicals were used in this study without further purification.

#### 2.1.1 Synthesis of Partially Deacetylated Chitin Nanocrystals (ChNCs)

Pure chitin and partially deacetylated chitin nanocrystals were extracted and purified as previously described with slight modification.^20,32^ Chitin nanocrystals (ChNC) were synthesized by an acid hydrolysis process using HCl (5 M) for 6 h at 90 °C and the ratio of solid to medium solution ratio was approximately (1/100). The nanocrystals were obtained after centrifugation at 7500 rpm for 30 min at room temperature. ChNCs were dialyzed using a cellulose membrane cut (12–14 kDa) for 1 week at room temperature using deionized water changed every 12 h until pH reached 4.5. The ChNCs were stored at 4 °C in a refrigerator until further use.

#### 2.1.2 Fabrication of the Hybrid LHM

Native insoluble wet acid tropocollagen (CO) was freeze-dried for 72 h to obtain 100 wt % dry material using the freeze-drying technique. One gram of dry CO was dispersed in 0.05 M hydrochloric acid at 0 °C for 48 h to obtain a homogeneous tropocollagen solution with high dispersibility. The swelled tropocollagen solution is homogenized at high speed (6 000 rpm) for 30 min at 0 °C. The highly dispersed solution obtained was placed in a Petri polypropylene plate to air-dry for 72 h at room temperature (15–17 °C) to obtain a native tropocollagen hydrogel membrane (CO). From our previous work, 1 wt % tropocollagen was selected for our study.^5^

CO-*g*-ChNCs-LHM was synthesized using a certain weight ratio of CO to ChNCs (1, 5, 10 wt %). The ChNCs were added dropwise to a slightly acidic CO medium (pH 4.5–5) at 0 °C to obtain a homogeneous mixture with high dispersibility of ChNCs without agglomeration. The resulting mixture of CO, ChNCs was agitated overnight at 4 °C to improve the assembly of the agitation and the high dispersibility of ChNCs in CO macromolecules. The dispersed solution is then cast into a polypropylene Petri dish and air-dried for 72 h at rt to obtain a layered material from the LHM assembly. The prepared samples were coded as mentioned in Table 1. In a water-aqueous solution, CO and ChNCs were cross-linked using a carbodiimide cross-linking system (EDC/NHS with a molar ratio of 2:1). After 3 h of cross-linking, CO-*g*-ChNCs-LHM was washed twice with 0.1 M Na_2_HPO_4_ and the fourth time with deionized water to remove byproducts.

**Table 1:** Composition of the LHM Materials Prepared^a^

Code	Abbreviations	CO	ChNCs	OCT	*V*_f_ (%)	CO/ChNCs/OCT ratio
I	Native CO	1	0	0	0	100
II	CO-g-ChNCs_1_	99	1	0	1.1	99/1
III	CO-g-ChNCs_5_	95	5	0	5.7	95/5
IV	CO-g-ChNCs_10_	90	10	0	11.4	90/10
V	CO-g-ChNCs_10_/OCT_0.1_	90	10	0.1	0.15	90/10/0.1
VI	CO-g-ChNCs_10_/OCT_1_	90	10	1	1.5	90/10/1
VII	CO-g-ChNCs_10_/OCT_2.5_	90	10	2.5	3.9	90/10/2.5

a CO = Collagen; ChNCs = Chitin nanocrystals; OCT = Octenidine dihydrochloride; *V*_f_ = Volume fraction.

A certain amount of Octenidine dihydrochloride (OCT) was added to the CO, ChNC mixture of 1 to 10% by weight. The OCT was dissolved in 1 mL (ethanol: water 1/1 v/v) and then added to the homogeneous mixture of CO/ChNC before the cross-linking step. The mixture was stirred for 5 h after adding OCT at 0 °C to obtain a homogeneous mixture with high dispersibility of OCT nanosphere drug without agglomeration. The resulting CO/ChNCs/OCT mixture was agitated overnight at 4 °C to improve the agitation and dispersibility of OCT in the CO/ChNCs matrix. The dispersed solution is then cast into a polypropylene Petri dish and air-dried for 72 h at room temperature to obtain an LHM assembly. The CO/ChNCs/OCT membrane was cross-linked, as described above.

### 2.2 Characterization of LHM

Attenuated total reflectance Fourier transform infrared spectroscopy (ATR-FTIR) was carried out using a Bruker Vertex V70 FTIR spectrometer and a Bruker Platinum ATR accessory with single reflection diamond crystal mount (Bruker Optik GmbH, Ettlingen, Germany). Samples were clamped directly against the diamond crystal using the platinum ATR sample clamp mechanism, ensuring consistent pressure per sample. Spectra were collected in the wavenumber region 3900–400 cm^–1^. Four data sets per sample were recorded, adding 128 interferograms per set. Spectra were measured at a scanner velocity of 40 kHz and a resolution of 4.0 cm^–1^. Using air as a reference, 128 background scans per sample were collected. Averaged spectra per sample were generated using Bruker OPUS version 7.2 software, where all spectra were corrected for ATR and vector normalized throughout the range. The second derivative spectra were calculated using a 13-point smoothing point Savitzky–Golay algorithm to better separate overlapping absorption bands within the Amide I band.

#### 2.2.1 Swelling Ratio

To determine the swelling ratio of the LHM, the membranes were cut into small pieces measuring 0.5 × 0.5 cm and weighed. Then, they were placed in glass vials containing solutions of water or phosphate-buffered saline (PBS) solutions and incubated at 37 °C. At regular intervals (1, 2, 4, 8, 12, 24, 48, 72 h). Native CO and LHM were removed and dried using filter paper to remove excess water or PBS from the hydrogel membrane surface. The percentage of swelling (%) of the LHM was then calculated using eq 1.^21,33^
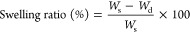
1where *W*_s_ is the weight of the swollen LHM, and *W*_d_ is the weight of the dry membrane; each value is averaged from three parallel measurements.

Tensile testing was used to investigate the strength of native CO, CO-g-ChNCs, and CO-g-ChNCs/OCT, which was necessary for sampling handling. It was carried out with Universal testing equipment Z010 from Zwick–Roell (Germany) and ASTM D5083 with a gauge length of 5 mm and a loading rate of 1 mm/min measuring cell. The samples are cut in a dog bone shape with a parallel specimen length of 12 mm. The testing rate was 10^–3^ s^–1^, and the tests were performed at laboratory temperature. The thickness of the samples was measured using SEM. Young modulus and tensile strength were calculated from the linear region of the stress–strain curves. The area calculated the toughness under the stress-strain curves.

The mechanical properties (tensile strength, young modulus, toughness, elongation at break) were based on average values of 5–10 samples. The synergistic properties of the hybrid hydrogel membrane were calculated from the following eq 2.^1,2,18,34,35^
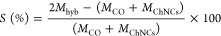
2where *M*_hyb_, *M*_CO_ and *M*_ChNCs_ represent the tensile strength, modulus, and toughness of the CO-g-ChNC hydrogel membrane, CO hydrogel membrane, and the ChNC membrane, respectively.

The morphology of native CO and CO-g-ChNCs and CO-g-ChNCs/OCT of the drug was visualized by transmission electron microscopy (TEM). The experiment was conducted with a Tecnai G2 spirit 12 electron microscope (FEI, Brno, Czech Republic). Native CO, CO-g-ChNCs, and CO-g-ChNCs/OCT were stained with uranyl acetate (UA) to increase the photos from the TEM contract. The staining agent was located on the surface of ChNCs, not on the surface of CO fibrils. The surface and cross-sectional morphology of the LHM were observed using a scanning electron microscope (SEM, KEYENCE, VE7800) at 3 kV. The LHM was fractured in liquid N_2_ and coated with an ultrathin layer of gold (10 nm) before being placed under the microscope. The specimens were stained with uranyl acetate in CO-g-ChNCs_10_ and CO-g-ChNCs10/OCT_2.5_. The staining agent was located on the surface of ChNCs, not on CO fibril surface. This explained why the dark and light bands of tropocollagen fibrils were not visualized compared to native CO fibrils, and the tropocollagen crossbanding was masked by the presence of these aggregates of ChNCs and OCTs@CO (Figure 3a, b).

*Rheological properties* of native collagen and collagen grafted with chitin nanocrystals in the presence and absence of octenidine dihydrochloride were investigated at rt and 37 °C using a rheometer. According to the preliminary results of the strain sweep test, native CO and the grafted hydrogel membrane were loaded into a parallel plate and subjected to a shear strain of 1% at a 0.5 mm gap under continuous oscillation. In the frequency mode, the storage moduli (*G*′) and loss moduli (*G*′′) of native and grafted hydrogel membranes were measured in the range of 0.1–100 rad/s at two different temperatures (rt and 37 °C).

X-ray photoelectron spectroscopy (XPS) was carried out with the Kratos Analytical Axis Ultra DLD system using a monochromatic Al Kα (*h*ν = 1486.7 eV) operating at 75 W (5 mA, 15 kV). Spectra were obtained using an analysis area of ∼300 × 700 μm. The Kratos charge neutralizer system was used for all analyses. The high-resolution spectra were measured with a step size of 0.1 eV and a pass energy of 20 eV. The instrument base pressure was 2 × 10^–8^ Pa. Spectra were analyzed using the CasaXPS software (version 2.3.15) by applying a Gaussian – Lawrence line shape for fitting and the ORIGIN 2016 software.

In vitro drug release kinetics. The experiment involved cutting the LHM into 1 mg pieces and placing them in centrifuge tubes with 10 mL of PBS. Tubes were incubated at room temperature and 37 °C while shaken at 100 rpm. At various time intervals (1, 2, 4, 8, 12, 24, 48, and 72 h), 50 μL of the release medium was removed and replaced with fresh PBS. The amount of OCT released was quantified using a UV–visible spectrophotometer at a wavelength of 281 nm (*R*^2^ = 0.99). A standard calibration curve determined the corresponding cumulative percentage of OCT released. The control of drug release measurement was carried out at 37 °C in an incubator with a shaking rate of 100 rpm for a specific time interval, and 50 μL of release medium (in PBS) was pipetted and replaced with fresh PBS. Subsequently, the vials were transferred to an incubator at room temperature. The amount of drug in the pulse release assay was determined using the same method to quantify cumulative OCT release. The cumulative percentage release of octenidine dihydrochloride was calculated using eq 3.^26,36^
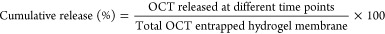
3

#### 2.2.2 Piezoelectric Measurements

Impedance spectroscopy was performed with Quatro Power Source (Novocontrol Technologies, Montabaur, Germany) and an E4991A RF analyzer (Agilent, Santa Clara, California, USA). Circular gold electrodes were deposited on both film sides with a Minilab 060 (Moorfield Technology, Knutsford, UK). The average thickness of the deposited gold electrode was measured to be 48 ± 2 nm using the in-built crystal balance. Their average diameter was 10.0 ± 0.1 mm, verified with the P-17 stylus profilometer (KLA, Milpitas, California). Two golden brass plates, each measuring 10 mm in diameter, served as contacts on the electrodes. Line and cell calibrations were performed before performing measurements. Each sample was measured five times in the frequency range 10^6^ – 3 × 10^9^ Hz, with an applied voltage of 0.1 V, in air and under controlled conditions of ∼22.0 ± 0.1 °C temperature and ∼55 ± 1% humidity level.

From impedance spectroscopy, it was possible to extrapolate and calibrate the data necessary for the estimation of the piezoelectric constant.^37^ Usually expressed as a matrix, in biopolymers the symmetry D∞(∞2), corresponds to an infinite cylindrical axis 38. It implies that the piezoelectric coefficient matrix is highly anisotropic and can be described by just two independent components *d*_14_ and *d*_25_. These components represent the degree of coupling between the mechanical stress and the electric field along the cylindrical and perpendicular directions, respectively. Moreover, *d*_25_ = −*d*_14_ thus the matrix representation was simplified to one single term using eq 4.
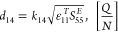
4where ε_11_^*T*^ is the product ε_0_*ε*_*r*_ of the vacuum permittivity (ε_0_) with the dielectric constant (*ε*_*r*_) at the resonance frequency (*f*_*R*_), *k*_14_ is the component of the piezoelectric coupling coefficient tensor, which represents the ratio of the induced electric charge in the *4* direction to the applied mechanical stress or strain in the *1* direction, and *S*_55_^*E*^ is a component of the elastic compliance tensor that describes the deformation response of a material to an applied stress.^38^ The element ε_11_^*T*^ can be extrapolated from the graph of the real part of the dielectric constant after finding *f*_*R*_ in the graph of the real part of the parallel impedance (*Z*_*p*_^′^), where it usually corresponds to a vertical asymptote.

#### 2.2.3 In Vitro Cell Culture

NHDFs were isolated from skin sections from plastic surgery with the Ethics Committee of the approval of the Olomouc University Hospital and the patient’s consent. The study was carried out according to the Ethics Code of the World Medical Association. The morphology and origin of the cells were authenticated in the Department of Histology, Palacky University Olomouc. NHDFs were cultured in Dulbecco’s modified eagle medium supplemented with 10% FBS (fetal bovine serum) and 1% penicillin-streptomycin under standard culture conditions (5% CO_2_, 37 °C). Cells were used between the second and third passages.^39,40^ The Saos-2 cell line was obtained from European Collection and Authenticated Cell Culture (ECACC) and cultivated according to the protocol in McCoys 5A (modified) medium supplemented with 10% FBS and 10% penicillin-streptomycin under standard culture conditions (5% CO_2_, 37 °C) standard culture conditions.^40^

The hydrogel membranes were cut in circles that fit into the 24-well plates. After 20 min of UV irradiation on both sides, the samples were hydrated with 500 μL serum-free culture medium for 24 h at 37 °C. Cells were seeded at a final concentration of 0.5 × 10^5^ cells per well, and cell viability was quantified after 1 day, 1 week and 3 weeks. After the incubation period, the medium was removed, and a serum-free medium supplemented with MTT (5 mg/mL) was applied to the cells for 2 h (37 °C, dark). The solution was removed and the crystals were dissolved again in DMSO with NH_3_ (1%, v/v). The absorbance was measured at a wavelength of 540 nm (Tecan, Czech Republic).^41^

SEM microscopy was provided to evaluate biocompatibility using a modified method by Schu et al.^42^ Cells were seeded in prewetted and UV-irradiated samples at a final concentration of 0.16 × 10^5^ cells per well and allowed to adhere for 24 h. Cells were fixed by rinsing three times in PBS buffer before adding 2.5% glutaraldehyde for 30 min. Following cell dehydration, the samples were dried with ethanol at different concentrations: 25, 40, 60, 80, 90 and 100%. Each concentration was incubated for 15 min. Immediately after 15 min of 100% ethanol, cells were incubated for 10 min with HMSD (hexamethyldisilazane).^42^

#### 2.2.4 Statistical Analysis

All data represented the mean ± standard deviation (SD). Statistical significance was determined using a one-way analysis of variance with Turkey’s test for multiple comparisons using OriginPro2020b (Originlab, Northampton, MA, USA).

## 3 Results and Discussion

### 3.1 Fabrication of LHM

**Figure 1:**
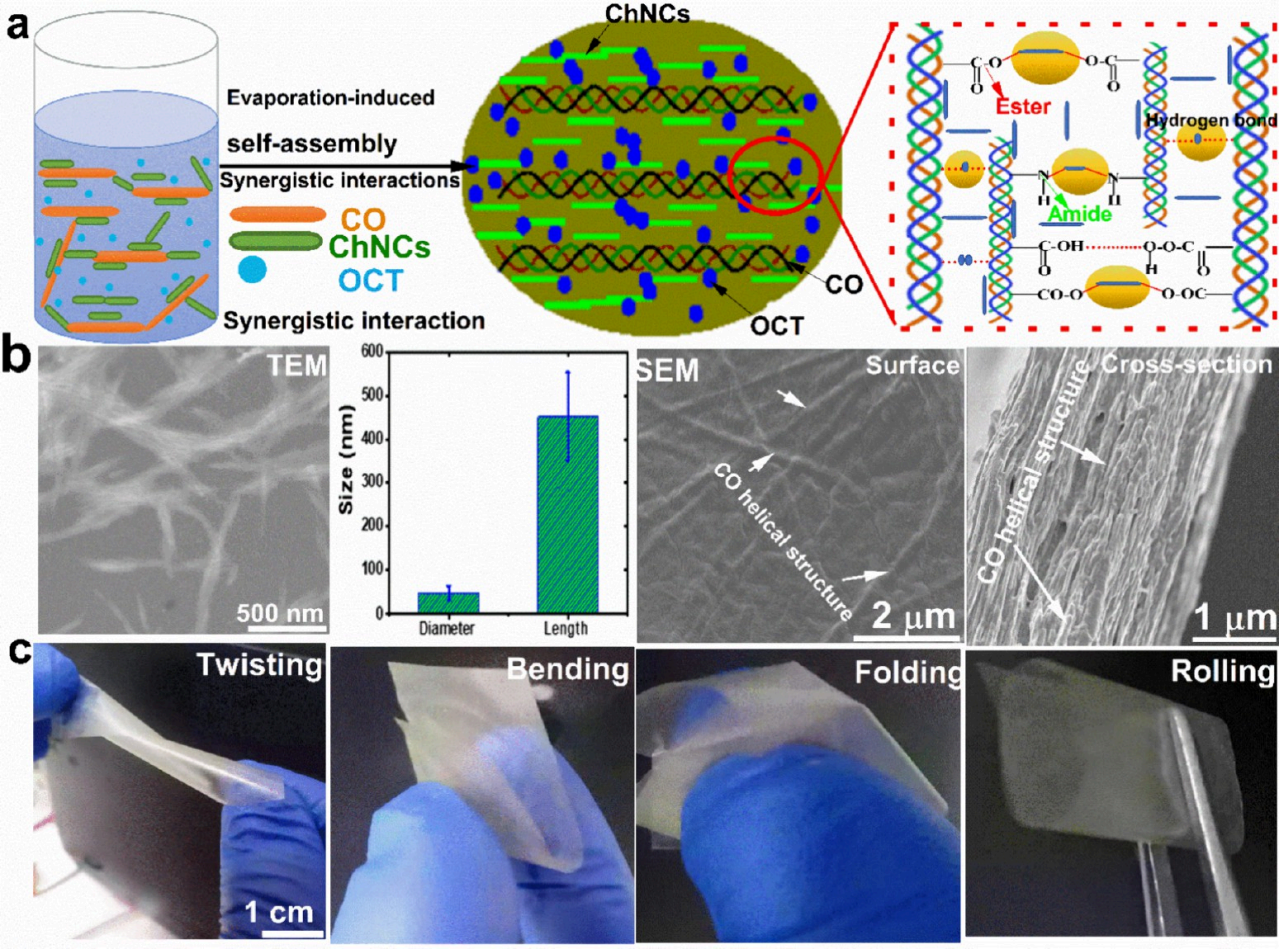
Aqueous dispersion of triple helical tropocollagen (CO), partially deacetylated chitin nanocrystals (ChNCs) and Octenidine dihydrochloride (OCT), which were assembled into artificial nacre by evaporation; proposed structural model for artificial LHM nacre, in which the CO, ChNCs and OCT network layer are alternatively stacked into a layered structure, anionic CO and cationic ChNCs were chemically (via ester/amide) and physically (hydrogen bonds, van der Waals force) in the presence of OCT (a); STEM of ChNCs, diameter/length of ChNCs, SEM of the surface and cross-section of native (arrows indicate helical CO fibrils). CO (b); digital photographs of the CO-g-ChNCs_10_/OCT_2.5_-LHM hydrogel membrane under arbitrary deformation showing a high level of transparency (c).

Triple-helical tropocollagen (CO) was dispersed in a slightly acidic HCl medium to obtain a well-dispersed solution of CO without denaturation (Figure 1a). Chitin nanocrystals (ChNCs) were prepared by acid hydrolysis of ChNCs with 27% DDA (%) and the DDA was confirmed by FTIR and ss-NMR.^20^ The resulting ChNCs exhibit a crystal diameter of approximately 45 ± 10 nm and a crystal length of approximately 400 ± 160 nm (Figure 1b). Never dried ChNCs were added dropwise to CO solution under slightly acidic conditions to prevent CO denaturation, ChNCs aggregation and improve the physical interaction of functional groups of both components at low stirring speed (100 rpm). Furthermore, the OCT suspension was slowly added to the mixture of CO/ChNC and then evaporated (Figure 1a).

The layered structure of hybrid nanocomposite membranes was proposed as shown in Figure 1a. The OCT nanosphere particles and ChNCs adhered to CO through covalent solid and hydrogen bonds between different functional groups of the three components (Figure 1a). Different weight ratios of CO and ChNCs (1/1, 1/5, 1/10) and coded as CO-g-ChNCs_1_, CO-g-ChNCs_5_, and CO-g-ChNCs_10_, respectively. The CO-g-ChNCs_10_ have been selected to study the effects of OCT on LHM performance properties. The different weight ratio of OCT was added to CO-g-ChNCs_10_ and coded as (CO-g-ChNCs_10_/OCT_0.1,_ CO-g-ChNCs_10_/OCT_1_ and CO-g-ChNCs_10_/OCT_2.5_). During evaporation process, the ChNCs were aligned into the CO helical structure macromolecule. The SEM of native CO (surface and cross section) shows the triple helical stricture of CO and the compact structure of native CO (Figure 1b).

Furthermore, the flexibility and mechanical properties of the LHM were tested. Figure 1c depicts the photographs of the LHM at various positions in the wet state with a high optical transmittance above 90%. The LHM could be twisted, rolled, bent, and folded without any damage under various arbitrary deformations. These results show the high flexibility of the LHM membrane. It has also been noted that the gel membrane regained its original shape after releasing these external stresses.

### 3.2 Morphological Properties

**Figure 2:**
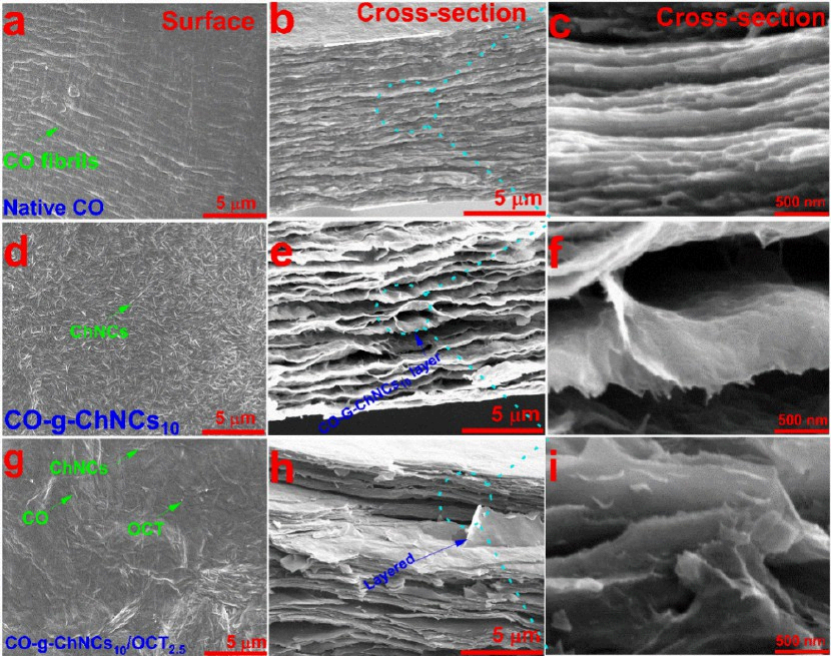
Surface and cross-sectional SEM images of CO (a–c), CO-g-ChNCs_10_ (d–f)_,_ and CO-g-ChNCs_10_/OCT_2.5_ (g–i).

Fractographic analysis was performed to investigate structural variables that influence the mechanical response of the material. Native CO exhibits brittle behavior (Figure 2a-c) while the CO-g-ChNCs_10_ membrane shows a different behavior, with many layers of materials being pulled during fracture (Figure 2d-f). The edges of the CO-g-ChNCs_10_ layers are curved rather than flat, indicating their deformation during crack propagation. This requires more deformation energy. The SEM micrograph in Figure 2f shows the detailed shape of the bent sheets at the nanometer scale.

After adding OCT nanosphere particles with a size of about 30–40 nm into the CO-g-ChNCs matrix, the scanning electron microscope (SEM) analysis reveals that all three components (CO, ChNCs, OCT) form a completely homogeneous layered sheet (Figure 2g-i). Furthermore, the edges of the CO-g-ChNCs_10_ sheet appear thinner and more strongly curved. This suggests that the addition of OCT influences the morphology of the CO-g-ChNCs_10_ sheets, potentially improving their mechanical properties (see Figures 8, 9). The fracture morphologies of CO-g-ChNCs_10_ and natural nacre-like layers are compared. In both cases, irregularly shaped platelets were pulled out (Figure 2g-i). However, sheets layered with tropocollagen/chitin nanocrystals exhibit a curved morphology because of their flexibility. This distinction in fracture morphology highlights the unique properties of CO-g-ChNCs layered sheets. The SEM photos indicated that the ChNCs increase the surface roughness of the hydrogel membrane compared with CO hydrogel membrane surface (Figure 2). The synergistic performance of interaction between CO and ChNCs and the potential for enhancing CO mechanical properties sheets through improved interfacial bonding and load transfer are highlighted.

**Figure 3:**
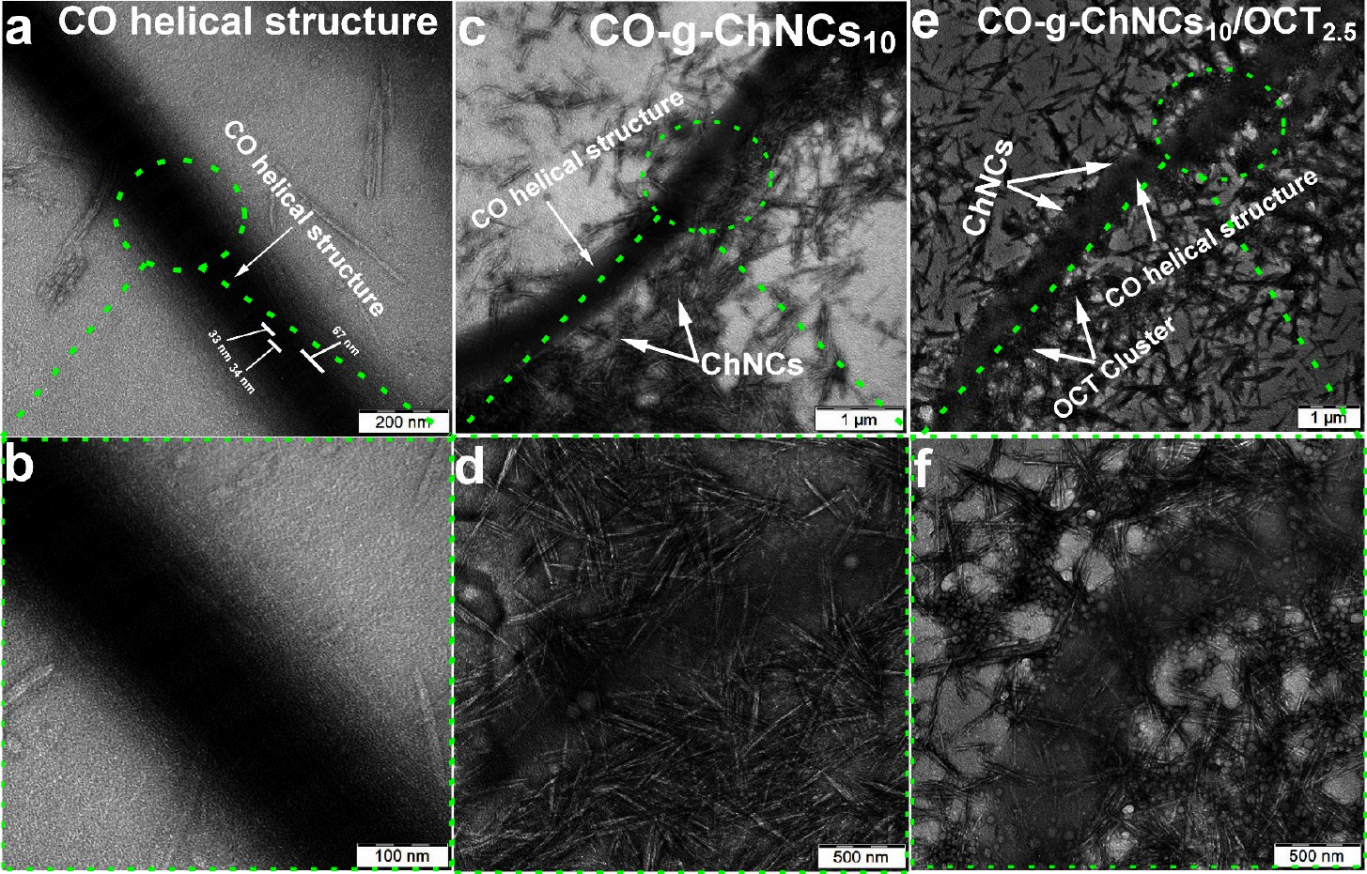
TEM images of native CO (a, b), CO-g-ChNCs_10_ (c, d), and CO-g-ChNCs_10_/OCT_2.5_ (e, f).

Figure 3 shows the high-resolution transmission electron microscope (HR-TEM) of native CO and CO-g-ChNCs_10_ and CO-g-ChNCs_10_/OCT_2.5_. Dispersion of CO in a slightly acidic medium did not affect the tropocollagen helical structure (Figure 3a, b). The dark band represents the “gap region” caused by a void between consecutive triple-helices; the light bar represents the overlap region between neighboring triple helices (Figure 3a). The TEM of CO-g-ChNCs_10_ shows the interactions between CO and ChNCs (Figure 3c, d) showing the chitin nanocrystal adhered to the CO fibrils. The TEM of CO-g-ChNCs_10_/OCT_2.5_ shows the OCT nanospheres attached to the CO fibrils in small clusters (Figure 3e, f). The OCT nanosphere protects the ChNCs from directly adhering to the CO surface (Figure 3e, f).

### 3.3 LHM Characterization

**Figure 4:**
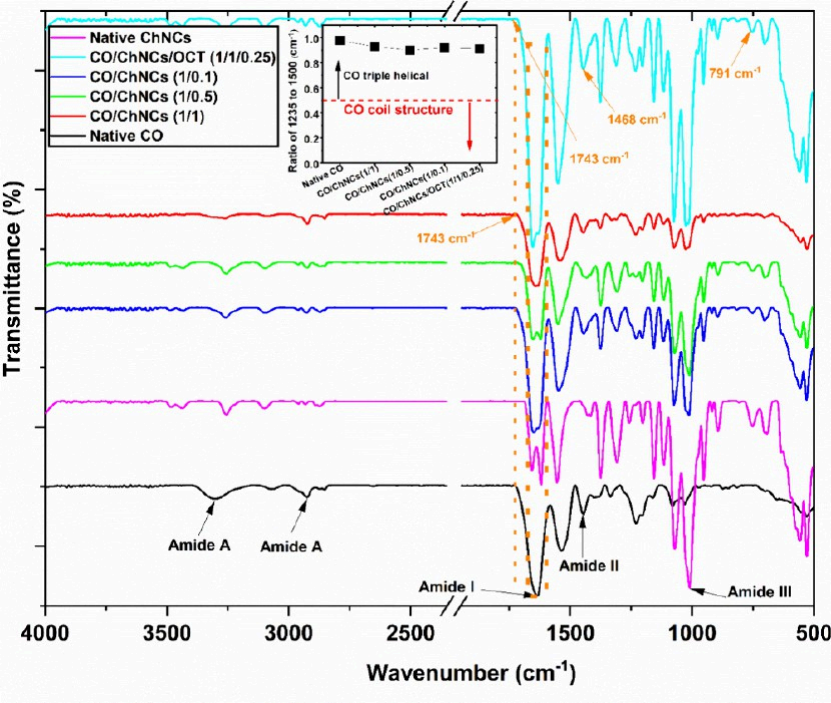
FTIR spectra of LHM.

To determine whether triple-helical tropocollagen material was present in the hydrogel membrane matrix, we analyzed the samples using ATR-FTIR (Figure 4). This method detects the absorbance from bond vibrations. It can describe the tertiary structure of tropocollagen macromolecules. Figure 4 compares the ATR-FTIR spectra for native CO, CO-g-ChNCs, and CO-g-ChNCs/OCT, showing apparent differences in the spectra. The native triple helical peaks at 3400–2900 cm^–1^, 1650 cm^–1^, 1550 cm^–1^, and 1400–1200 cm^–1^ for amide A, amide I, II, and III, respectively.^43,44^ The amide I peak was mainly associated with the C=O stretching vibration and was directly related to the backbone conformation. The Amide II is due to N–H/C-N bending/stretching vibrations in a triple-helical tropocollagen macromolecule. The Amide III was a very complex and sharp band, depending on the nature of the side chains and hydrogen bonding, and therefore was only of limited use to extract structural information.^43,45^

In this study, the integrity of the tropocollagen triple helix was evaluated considering the maximum absorbance ratio of the Amide III (1235 cm^–1^) and the 1450 cm^–1^ band corresponding to the stereochemistry of the pyrrolidine rings of the proline and hydroxyproline residues,^8^ essential for the triple helix conformation. The triple-helix tropocollagen conformation was intact if this ratio is close to 1.0, while the ratio values for denatured tropocollagen are around 0.5.^45,46^ The triple helical of CO was constant after adding different percentages of ChNCs (up to 95%; Figure 4). Due to the overlap between functional groups of tropocollagen (amino, amide, carboxylic, hydroxyl) and chitin nanocrystal groups (−NH_2_, −NHCOCH_3_, −OH), it was not easy to quantify the new amidation and esterification bond between both components. Small peaks were observed at 1743, 1468, and 791 cm^–1^, which were attributed to NH bending and rocking of the ester and amide, respectively, indicating the successful coupling of the amide by the EDC/NHS coupling agent.

**Figure 5:**
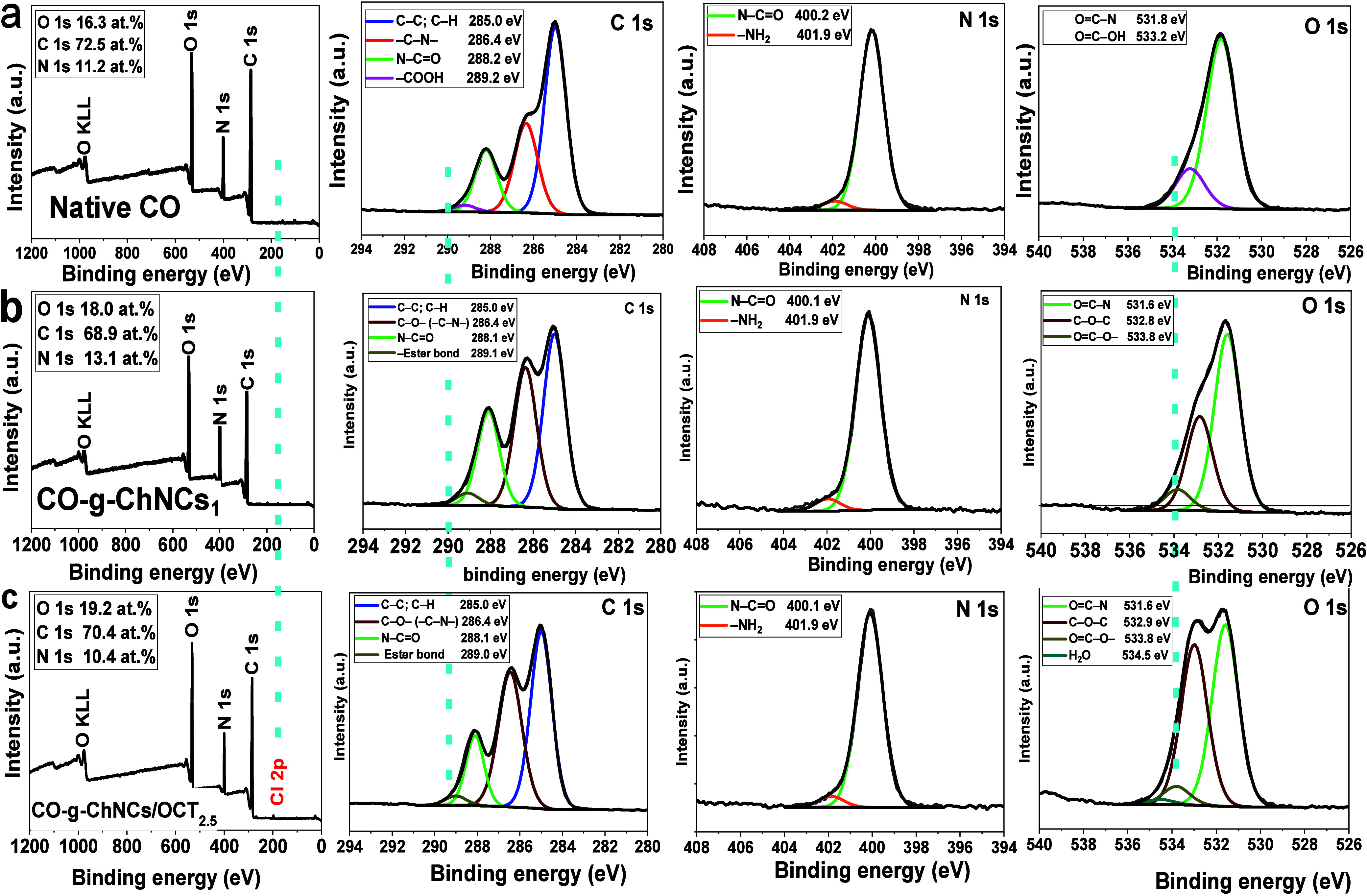
XPS spectra of native CO (a); CO-g-ChNCs_10_ (b); CO-g-ChNCs_10_/OCT_2.5_ (c).

XPS analysis was used to examine the changes in the chemical environment of the elements to investigate the interactions between the CO-g-ChNCs and CO-g-ChNCs/OCT at the molecular level. Figure 5 shows the XPS of CO-g-ChNCs_10_ and CO-g-ChNCs_10_/OCT_2.5_. The broad spectrum of all samples shows signals of only peaks of elements O 1s, C 1s, and N 1s, except CO-g-ChNCs_10_/OCT_2.5,_ which shows one more Cl 2p peak related to chloride atoms of the OCT drug. In a comparison of native CO before and after cross-linking (Figure 5a, S1), there were no significant differences in the XPS shift obtained before and after cross-linking the tropocollagen hydrogel, confirming that the EDC-NHS cross-linking agent cannot enter the gap between tropocollagen molecules in microfibrils. Furthermore, we confirm that the D-periodic banding pattern of tropocollagen fibrils is not altered by using the EDC/NHS cross-linker agent and that there is no denaturation during tropocollagen cross-linking with EDC/NHS. In particular, the intensities of the native cross-linked CO hydrogel O 1s O—C=O— and N 1s −NH_2_ were decreased after cross-linking the CO hydrogel membrane with EDC/NHS. The O—C=O— area (%) decreased from 23.5 to 18.9%, indicating more selective esterification than the amidation reaction.^47^

In CO g-ChNCs (Figure 5b), the C 1s spectra of CO and ChNCs could be deconvoluted into four peaks of 285.0, 286.4, 288.1, and 289.1 eV that belong to C–C/CH, C–O/C–OH/C–N, N—C=O and O—C=O— ester bonds. Compared to C 1s- COO^–^ of the native CO hydrogel (289. Two eV), the intensity of CO–COO^–^ of CO-g-ChNCs was shifted to 288.1 eV. In the O 1s spectrum, the new peak appeared at 533.8 eV after the chemical modification of CO with ChNCs, indicating not only the chemical interaction between CO and ChNCs but the physical bonding. The new peak indicated that the chemical interaction between CO and ChNCs was not only physical bonding. This new peak is due to the chemically boned bonds between CO and ChNCs through amidation/esterification reactions. Furthermore, the binding energy of the component at 288.1 eV of N–C=O does not change after modification with ChNCs loaded with the OCT drug, indicating the unbroken triple helical conformation of the tropocollagen macromolecule with the introduction of ChNCs. No significant differences were visualized in the CO-g-ChNCs_10_/OCT_2.5_ spectrum compared to those observed for the CO-g-ChNCs_10_ hydrogel membrane (Figure 5c), indicating that the binding between the CO-g-ChNCs_10_ hydrogel membrane matrix and the OCT drug was physically bonded only.

### 3.4 Rheological Behavior

**Figure 6:**
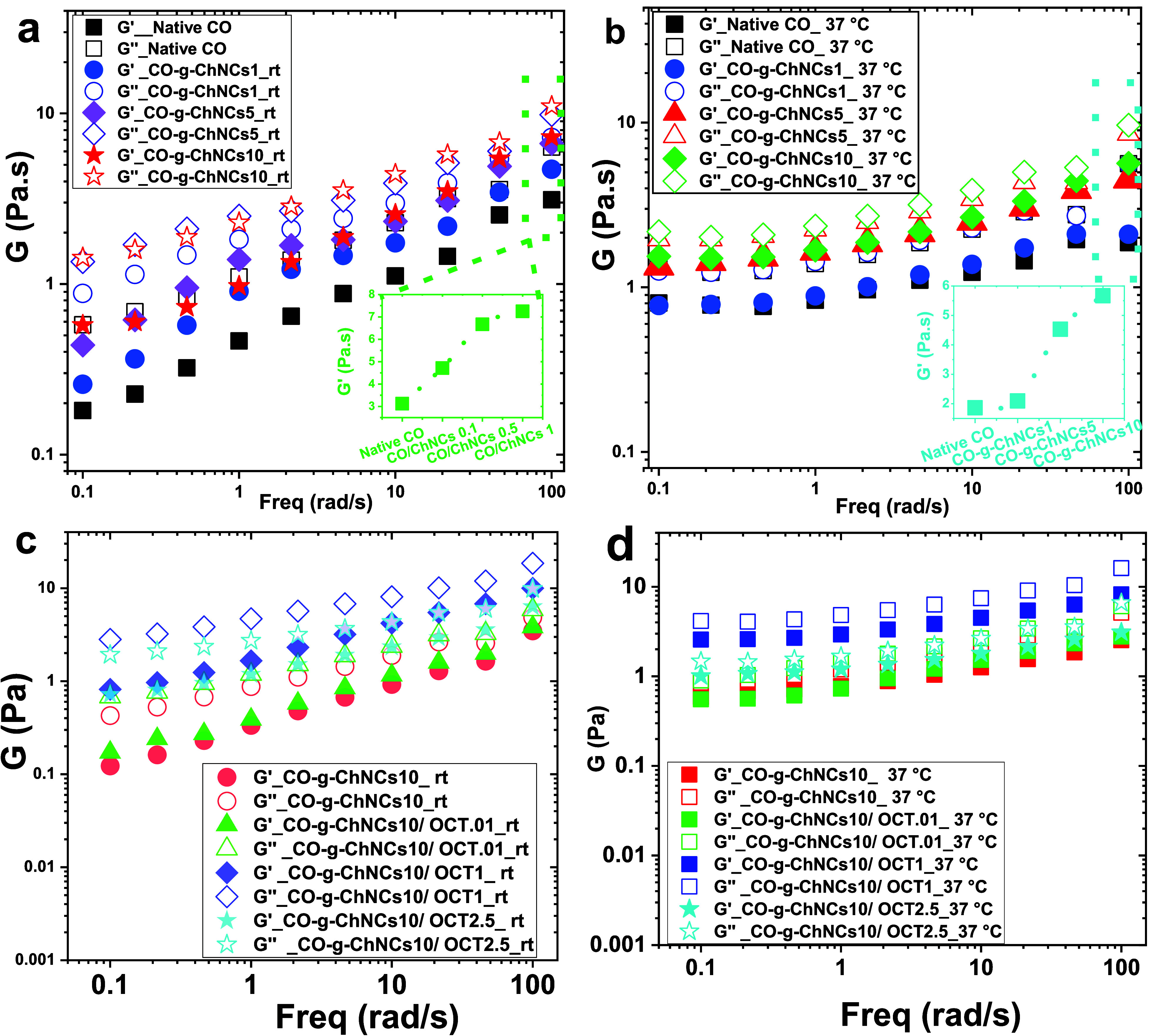
Rheological properties of native CO, CO-g-ChNCs (different ChNCs content) at rt (a) and 37 °C (b). CO-g-ChNCs/OCT (different OCT content) at rt (c) and 37 °C (d).

The rheological results of the CO-g-ChNCs solution with various loads of ChNCs are shown in Figure 6 at both temperatures (rt, 37 °C). The first observation is that *G*′′ was more significant than *G*′ for pure CO and CO-g-ChNCs, indicating that these solutions behaved as elastic liquids. As shown in Figures 6a, b, the *G*′ modulus of CO-g-ChNCs increased with increasing doses of ChNCs (1 to 10 wt %). Under the frequency of 100 rad s^–1^, the *G*′ of native CO was 3.1 Pa, and the *G*′ of the CO-g-ChNCs with ChNCs of 10 wt % was 7.3 Pa, almost twice higher. The interactions between ChNCs and CO via hydrogen bonds that act as physical cross-links formed a cross-linked network in the CO and ChNCs matrix, leading to the increasing mechanical properties of the CO-g-ChNCs hydrogel membrane. The increase in *G*′ also reflects the increase in the CO cross-linking density. The loss modulus *G*′′ also increased with increasing loading of ChNCs, and it was insensitive to frequency. No crossover of *G*′′ at low frequencies was observed, a characteristic of a cross-linked network (Figure 5a, b) at rt and 37 °C. Figures 6c, d show the rheological properties of CO-g-ChNCs_10_/OCT (0.1, 1, 2.5 wt %) at different temperatures (rt, 37 °C). The *G*′ modulus of CO-g-ChNCs increased with the increasing ratio of OCT (0.1, 1, 2.5 wt %). CO-g-ChNCs10/OCT0.1 shows a high *G*′ compared to CO-g-ChNCs10/OCT2.5 at rt and 37 °C (Figure 6 c, d). Due to the high OCT ratio generated by the cluster system interacting with the CO-g-ChNC matrix (Figure 3e, f).

### 3.5 Swelling Behavior

**Figure 7:**
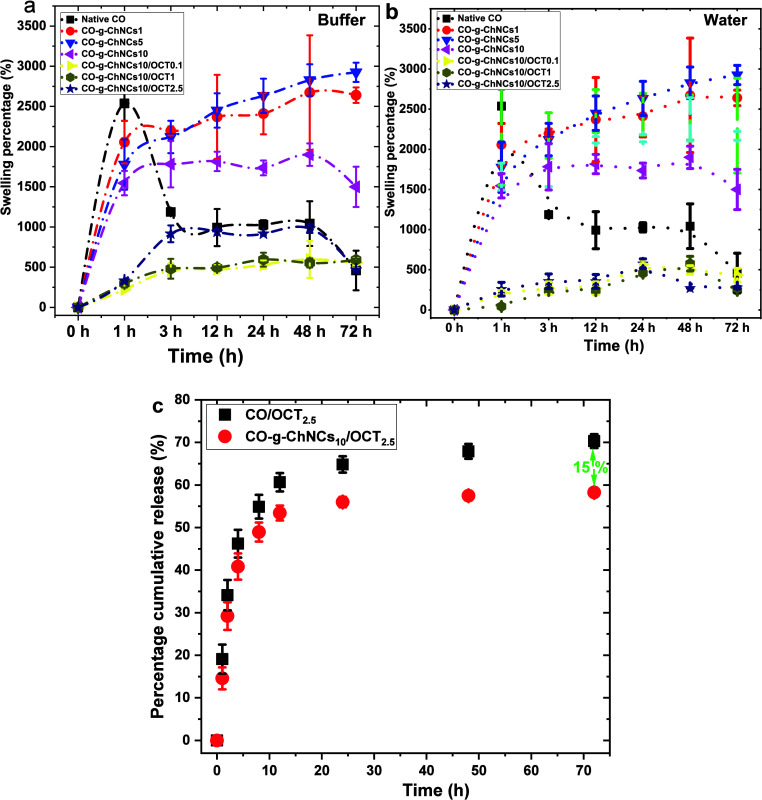
Swelling ratio in buffer (a); in water at 37 °C (b); *in vitro* cumulative percentage of OCT release from LHM (c).

Figure 7 represents the swelling properties of native CO and CO-g-ChNCs with different ratios of ChNCs and OCT. In the first hour, CO shows a high percentage of absorbing (%) in both water and PBS compared to the dry weight of the tropocollagen membrane due to the increased hydrophilicity of the tropocollagen macromolecule in both water and PBS medium (Figure 7a, b). The percentage of swelling of native CO decreased significantly after 1 h of immersion in both PBS and water (Figure 7a, b). The addition of different ChNC ratios increased the swelling percentage compared to CO after 3 h of immersion in PBS and water solution medium. The maximum swelling percentage was visualized on the CO-g-ChNCs_5_ hydrogel membrane in a buffer and water medium (Figure 7a, b). With the addition of OCT, the swelling rate of the LHM decreased with increasing OCT ratio of OCT in the matrix. It was lower than the native CO hydrogel membrane, but still showed an acceptable value above 500% compared to the dry hydrogel membrane (Figure 7a, b).

The OCT-loaded hydrogel membrane depot was covered by adding PBS buffer, and a slight shake initiated the release of OCT. As shown in Figure 7c, the release of OCT from both formulations was controlled and presented a controlled release. In particular, the initial burst from the hydrogel membrane was controlled, indicating that the OCT nanosphere was effectively loaded into the CO-g-ChNCs hydrogel matrix. The extent of OCT release control was low in the CO-g-ChNC hydrogel membrane, implying that the OCT released from CO hydrogel membrane further interacted with ChNCs and delayed the release of OCT. The 55% release of OCT from the CO-g-ChNCs hydrogels was observed 3 days after incubation in PBS. On the contrary, 70% of OCT was released from the native CO hydrogel membrane, indicating that the ChNCs reinforcement in the LHM networks controlled the release pattern.

### 3.6 Mechanical Properties of LHM

**Figure 8:**
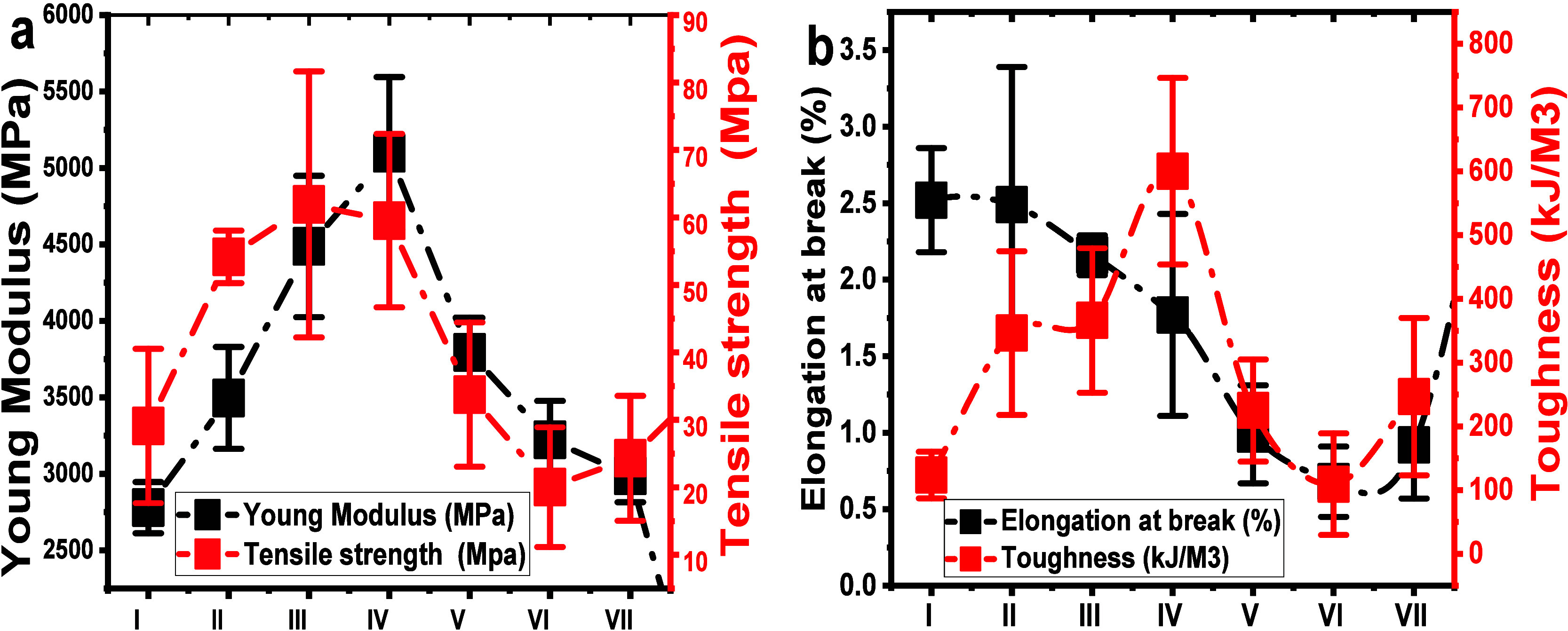
Mechanical properties of CO and CO-g-ChNCs/OCT–LHM Modulus and strength (a); Toughness and elongation at break (b).

The mechanical properties of the CO hydrogel membrane are crucial for applications related to tissue regeneration purposes. Figure 8 shows the effects of different ratios of ChNCs on the mechanical properties, specifically modulus, toughness, elongation at break, and strength of the LHM. The native CO membrane exhibited a modulus of 2500 MPa. However, after adding nanofiller chitin nanocrystals, the modulus was significantly increased as the amount of ChNCs (1 to 10 wt %) increased, reaching 5300 MPa (110% increase) (Figure 8a). The tensile strength of the hydrogel membrane showed notable improvements with an increasing ratio of nanocrystals added to a solution of CO triple-helical structure.

The highest modulus and strength values were obtained in a CO-g-ChNCs_10_ ratio (as depicted in Figure 8a). Although the modulus and strength of the hydrogel membrane slightly decreased with an increase in the percentage of OCT nanospheres in the CO/ChNCs matrix, they remained higher than that of CO hydrogel membrane. Figure 8b focuses on the toughness and elongation at break (%) of LHM. The native hydrogel membrane exhibited a high elongation at break (EP) value. However, upon the addition of ChNCs and nanosphere drug, the EP decreased slightly compared with that of native CO hydrogel membrane, although the decrease was insignificant. The toughness properties of the LHM increased significantly after adding different ratios of ChNCs (1 to 10 wt %). They decreased slightly with the introduction of the OCT nanospheres into the LHM (Figure 8b).

**Figure 9:**
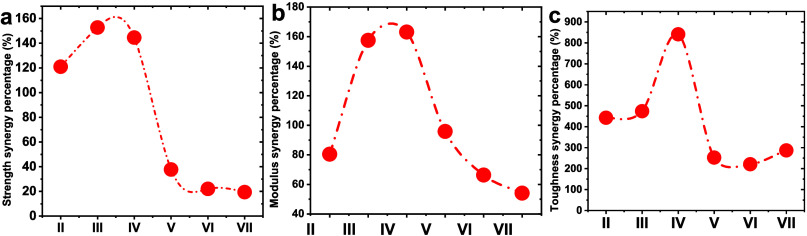
Synergy percentage of LHM increases with ChNCs content in grafted CO-g-ChNCs-LHM. Modulus synergy percentage (a); Strength synergy percentage (b); Strength synergy percentage (c). I = native CO; II = CO-g-ChNCs_1_; III = CO-g-ChNCs_5_; IV = CO-g-ChNCs_10;_ V = CO-g-ChNCs_10_/OCT0_.1_; VI = CO-g-ChNCs_10_/OCT_1_; VII = CO-g-ChNCs_10_/OCT_2.5_.

The synergistic effects of the building blocks of triple helical tropocollagen and partially deacetylated chitin nanocrystals on improving mechanical properties can be quantified by the percentage of synergy, as shown in Figure 9a-c. The rate of strength synergy of CO-g-ChNCs increases with increasing ChNCs content (1 to 10 wt %) and reaches a maximum value of 152.7% for CO-g-ChNCs_5_ (Figure 9a). Meanwhile, the percentage of modulus synergy also gets the maximum value of 163.08% for CO-g-ChNCs_10_ in the hydrogel membrane matrix, indicating that the synergistic effect can be optimized and adjusted with the change in the ChNCs ratio in the matrix (Figure 9b). The percentage of toughness synergy also increases with increasing ChNCs and reaches a maximum of 840.75% for CO-g-ChNCs_10_ (Figure 9c). The addition of nanosphere OCT drug decreased the synergistic performance between CO and the ChNCs; the OCT nanospheres generated some cluster structure that inhabits the direct inceration between CO and the ChNCs. The same behavior was investigated by adding nanosiliate (SiO_2_) to the acrylamide-based hydrogel.^48−50^

Furthermore, the percentage of synergy can be further enhanced through strong covalently cross-linking interface interactions and the construction of different types of hydrogen bonds between CO and ChNCs, as shown in Figures 4 and Figure 6a, which also provides a new strategy for improving the mechanical properties of nanocomposites-layered hydrogel membranes.^3^

**Figure 10:**
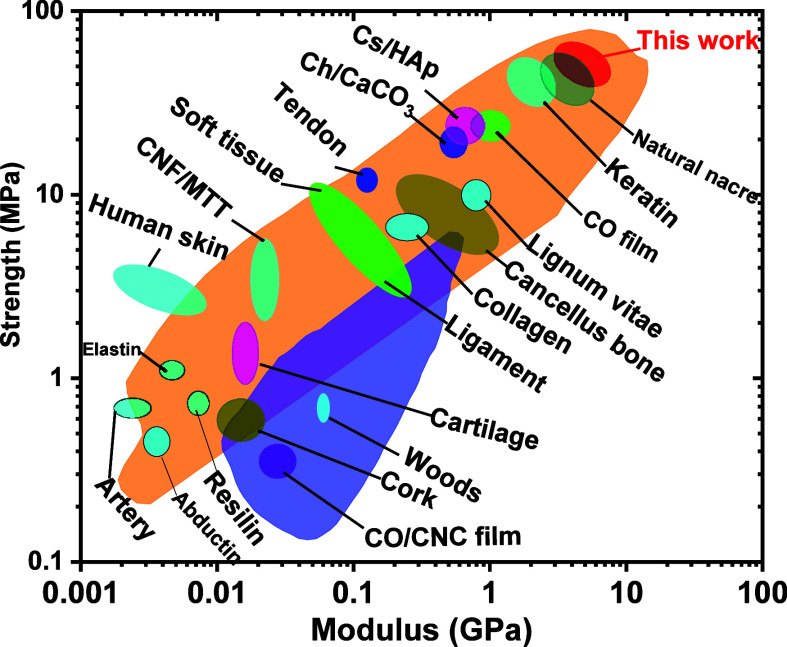
Ashby plot graph of the comparison of mechanical properties of our nacre-like NPs-type of CO-g-ChNCs_10_/OCT_2.5_-LHM with natural nacre and prepared layered CO/polymer composite.

The mechanical performance of our nacre-like layers with those of natural nacre and the reported layered composite material with a higher nanofiller chitin content is shown in Figure 10. Our LHM was stronger than cancellous bone,^1,51^ cartilage,^52,53^ human skin,^54^ a few artificial nacre materials^34,54−56^ and synthetic composites such as CS/HAP,^57,58^ Ch/CaCO_3_^59−61^ and CNF/MTT.^18^ As discussed above, the LHM compounds synthesized in this study displayed multilevel controllable hierarchical structures.

In conclusion, the addition of ChNCs and OCT nanospheres influenced the mechanical properties of CO hydrogel membrane by enhancing its modulus, toughness, and strength, but potentially reducing its elongation at break. These findings are essential to tailor the mechanical characteristics of tropocollagen hydrogel membranes for specific tissue regeneration applications.

### 3.7 Piezoelectric Properties of LHM

**Figure 11:**
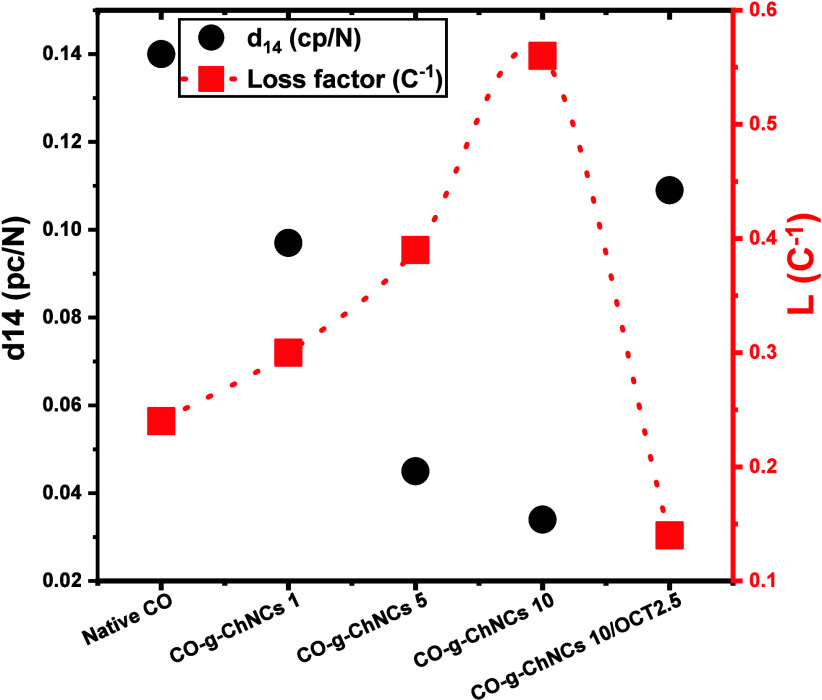
Plot of the piezoelectric tensor element *d*_14_ and piezoelectric loss *L*.

The dielectric analyses performed with the impedance spectroscopy provided us with the value of piezoelectric constant and loss useful to predict the performance as cell growth scaffold (Figure 11). They revealed a quite high value of *d*_14_ for the native CO hydrogel membrane of 0.14 pC/N. This value was much higher than the literature value, where the highest value found was 0.102 pC/N,^38^ while other studies claimed much lower values: 0.057 pC/N^62^ and 0.036 pC/N.^37^ The high value of *d*_14_ could be attributed to the higher stiffness and compactness of the piezoelectric sites as a consequence of of the well-preserved helical structure of the prepared hydrogel membrane.

The introduction of ChCNs decreased as 14 logarithmically An analogous decrease in the piezoelectricity of the native CO nanocomposite was observed in the literature by adding hydroxyapatite^61^ and natural rubber^18^ to native CO. This behavior was attributed to the warping deformation caused by the extraneous element within the triple-helical tropocollagen macromolecules, dependent on the geometry of the filler rather than on its nature. Fitting the function of the collagene concentration and fitting it with an exponential growth equation, it was possible to extrapolate the theoretical value of 14 for the pure ChNCs, which would be approximately 0.036 pC/N (Figure S4). In parallel, the addition of ChNCs to collagen composites resulted in a significant increase in L, which was directly proportional to the concentration of collagen. This increase in L suggests that any energy missing due to a lower piezoelectric effect in the composites is dissipated by the system either through heat or by moving the nanocrystals, leading to faster degradation of the material.

The addition of OCT conveyed a noticeable increase in d_14_, almost nullifying the negative impact of the nanofiller, even though CO concentration was even lower. This effect was attributed to the more homogeneous dispersion of the chitin nanocrystals evidenced by the morphological analyzes (Figure 2, 3). The ChCNs clusters were much smaller and better oriented under sheer stress during membrane preparation, resulting in a smaller warping impact on the collagen structure (Figure 3e, f). This behavior was visible in the piezoelectric loss results as well. The OTC surfactant decreased *L* even under the value of the pristine collagen. The finer dispersion of the ChNCs in the collagen matrix sank the resistance that the larger clusters were nonhomogeneously dispersed opposed to the mechanical deformation of the matrix during the performance of the piezoelectric phenomenon. Moreover, in the presence of OTC, a synergistic effect between the filler and the matrix justifies a lower dissipation than in the pristine matrix. Such a synergy with effects on the mechanical behavior was also suggested by the elongation tests (Figures 8, 9).

The curing of the polymer did not significantly affect the piezoelectric loss of tropocollagen (cap L). This suggests that the increase in stiffness and compactness resulting from the curing process led to rasterized *d*_14_ without any negative effects of ChNCs on CO compounds, resulting in a significant increase in L, which was directly proportional to the tropocollagen concentration. This increase in L suggests that any energy missing from a lower piezoelectric effect in the composites is dissipated by the system either through heat or by moving the nanocrystals. The OTC decreases *L* even under the value of pure native CO (Figure 11). The finer dispersion of the ChNCs in the CO matrix sinks the resistance that more giant clusters are nonhomogeneous dispersed as opposed to mechanical deformation of the matrix during the piezoelectric phenomenon. Moreover, in the presence of OTC, a synergistic effect appears between the filler and the matrix to justify a lower dissipation than in the native matrix (CO/ChNCs). Another hypothesis is that OTC acts not only on the ChNCs dispersion but also on the CO morphology.

### 3.8 Cytotoxicity and Cell Adhesion Properties

**Figure 12:**
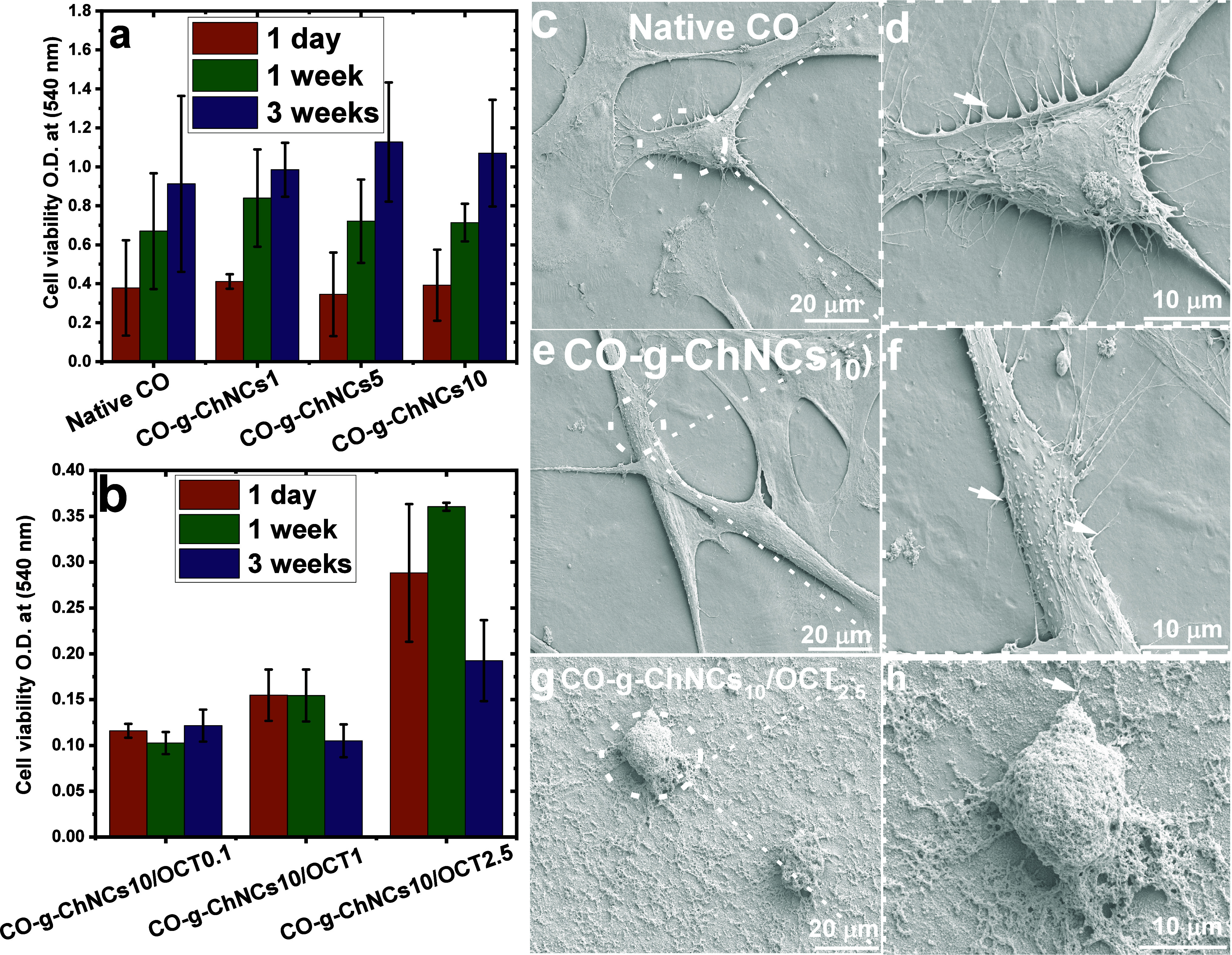
Cytocompatibility of native CO, CO-g-ChNCs, and CO-g-ChNCs/OCT after seeding with *NHDF* cells for 1 day, 1 week, and 3 weeks (a, b). SEM of native CO (c, d); CO-g-ChNCs_10_ (e, f); and CO-g-ChNCs_10_/OCT_2.5_ (g, h). The white arrow indicates the filopodia. Scale bars for (c, e, g) were 20 μm, and for (d, f, h) were 10 μm. All samples were measured three times to calculate the standard division (n ± 3).

This study investigated how the composition and assembly of triple-helical tropocollagen/nanocrystal chitin and drug compounds influence the growth of *NHDF* and Saos-2 cells (Figure 12, S2). Cells proliferated on the native CO, CO-g-ChNCs, and CO-g-ChNCs/OCT nanocomposite hydrogel membrane and were determined after 1, 7, and 21 days by the MTT assay. Cell viability increased with different ratios of chitin nanocrystals (1 to 10 wt %) after all incubation periods (Figure 12a). The cell viability of the CO-g-ChNCs/OCT hydrogel membrane was slightly decreased compared to different ratios of native CO and CO-g-ChNCs (Figure 12b). The behavior was also observed with Saos-2 cells by introducing the nanosphere OCT drug. The decrease in cell viability in the presence of OCT is due to the fast release of OCT (nonbonded OCT) from the CO/CHNCs matrix in the first 72 h. From the literature, OCT shows cytotoxic properties against different cell types (*NHDF, Saso2*) under certain concentrations.^64,65^ Cell viability decreased slightly compared to CO and CO-g-ChNCs with different ChNCs ratios (Figure S2a, b). The cell structure was preserved without visible breakage and basic details of the adhered cells were observed.

SEM examined the morphology of the *NHDF* cells on the hydrogel membranes. Figures 12c-f shows the SEM micrographs of *NHDF* on the nanocomposite hydrogel membrane after 24 h of culture. Cells grown on native CO and CO-g-ChNCs_10_ have typical fibroblast morphology with filopodia (Figure 12c-f). Cells grown in CO-g-ChNCs/OCT_2.5_ were more rounded and lacked the classical stretched structure (Figure 12g, h, Figure S3). Osteoblast cells (Saos-2) (Figure S2c-f) cultured on the nanocomposite hydrogel membranes connected by discrete filopodia formed an entangled network. From the enlarged magnification (Figure S 2d, f), the Saos-2 cells spread on the surface of the hydrogel membrane, and the number of cells increased as the ChNCs ratio in the matrix increased (Figure S3). From Figure S2c-f and Figure S3, NHDF and Saos-2 exhibited an elongated shape. They were anchored onto the surface of the nanocomposite hydrogel membrane by discrete filopodia, indicating the excellent adhesive performance of the hydrogel membrane.

No obvious cell spreading of both *NHDF* and Saos-2 was observed in CO-g-ChNCs/OCT_2.5_, probably due to two reasons, first: drastic swelling of CO-g-ChNCs/OCT_2.5_ (more hydrophobic like in Figure 7) in salty culture medium and the resultant shrinkage during drying, along with deformation or detachment of adherent cells, second: The presence of OCT could prevent the well adhesion of cells to the LHM surface.

Chitin has been confirmed to support the initial attachment and spread of *NHDF* and *Saos2*.^27,63^ On the other hand, chitin nanocrystals could increase the surface roughness of the layered nanocomposite hydrogel membrane, as visualized in Figure 2. Chitin nanocrystals (ChNCs) were able to promote cell adhesion.^27,28^ Furthermore, the hydrophilicity of partially deacetylated chitin and functional groups such as-NH_2_ was supposed to facilitate effective calcium phosphate deposition^64,65^ and the formation of an apatite layer, which can further promote osteoconductivity.^66,67^ and anti-inflammatory properties can prompt skin healing. Therefore, the introduction of chitin nanocrystals significantly improved the LHM affinity and cytocompatibility of NHDF and Saos-2 cells, resulting in a significant potential in scaffold materials for the regeneration of skin and bone tissue and control drug release applications.

## 4 Conclusions

In this study, a novel method for creating a layered nacre-like material has been introduced, utilizing triple-helical tropocollagen (CO) and partially deacetylated chitin nanocrystals (ChNCs), along with octenidine dihydrochloride nanosphere particles (OCT). ChNCs play a crucial role in enhancing the stability of the triple-helical structure of tropocollagen and promoting fibrillar arrangement through hydrogen bonding and other weaker electrostatic interactions. This hierarchical microstructure formation resulted in the development of a layer material, referred to as LHM. The addition of ChNCs and OCT nanospheres influenced the mechanical properties of the CO hydrogel membrane by enhancing its modulus, toughness, and strength, but potentially reducing its elongation at break.

The LHM exhibited remarkable improvements in mechanical properties, including increased modulus, strength, and toughness, compared to the LHMs prepared without ChNCs. These enhancements were attributed to the incorporation of ChNCs and OCT, a drug model. The synergy between ChNCs and OCT further contributed to the enhanced mechanical performance of the material, surpassing that of natural nacre and other synthetic layered composite materials. A notable aspect of the synthesized nacre-like material was its excellent biocompatibility, demonstrated by its ability to support the adhesion, spread and proliferation of *NHDF* and *Saos-2* cells. By varying the ratios of ChNCs, the material properties may be tailored to meet specific requirements. Overall, this study presented a promising high-performance layered nanocomposite material that combines the strengths of protein and polymer nanocrystalline chitin. The LHM material exhibited outstanding mechanical properties and biocompatibility and holds great potential for a wide range of applications, in skin/bone tissue engineering, drug delivery systems and potentially more.

## Supporting Information Available

Figures S1–S4 contain additional characteristics and properties of the prepared hydrogel membrane materials (DOCX)
