# Supplementary material for: Self-Assembled Hydrogel Membranes with Structurally Tunable Mechanical and Biological Properties
Source: Biomacromolecules. 2024 May 13;25(6):3449–63. doi: 10.1021/acs.biomac.4c00082 (PMC11170955; doi:10.1021/acs.biomac.4c00082)
Supplement: Supplementary file 1 — bm4c00082_si_001.docx [file bm4c00082_si_001.docx]

**Supporting information**

Self-assembled hydrogel membranes with structurally tunable mechanical and biological properties

Rasha M. Abdel-Rahman^a,b^, A. M. Abdel-Mohsen^a,b,c*^, Jana Frankova^d^, Francesco Piana^b^, Lukas Kalina^f^, Veronika Gajdosova^b^, Ludmila Kapralkova^b^, Muhammed Arshad Thottappali^b^, Josef Jancar^a,e^

**^a^** CEITEC-Central European Institute of Technology, Brno University of Technology,

Purkyňova 656/123, Brno 61200, Czech Republic

**^b^** Institute of Macromolecular Chemistry, Czech Academy of Sciences, Heyrovského nám. 2,

Praha 162 06, Czech Republic

**^c^** Pretreatment and Finishing of Cellulosic Based Textiles Department, Textile Industries

Research Institute, National Research Centre, 33 EL Buhouth St., Dokki, Giza 12622, Egypt

**^d^** Department of Medical Chemistry and Biochemistry, Faculty of Medicine and Dentistry,

Palacký University, Hněvotínská, 3, 775 15, Olomouc, Czech Republic

**^e^** Faculty of Chemistry, Materials Research Centre, Brno University of Technology, Purkyňova

464/118, Brno 61200, Czech Republic

Corresponding authors:

*CEITEC-Central European Institute of Technology, Brno University of Technology, Purkyňova 656/123, Brno 61200, Czech Republic: e-mail**:** [abdel-mohsen@ceitec.vutbr.cz](mailto:abdel-mohsen@ceitec.vutbr.cz)

**Fig. S1.**  XPS of native CO before crosslink (a) and native nanocrystals chitin membrane (b).

The broad spectrum of native chitin film shows only three element signals: O 1s, C 1s, and N 1s peaks. C 1s was deconvoluted to three peaks at 285.0, 286.6 and 288.2 eV related to C-C/C-H, C-O/C-OH, and N-C=O, respectively. In the N 1s spectrum, two peaks appeared at 400.0 and 401.5 eV related to N-C=O and NH_2_ groups. For the O 1s spectrum, two peaks were deconvoluted at 531.6, and 533.0 eV corresponding to O=C-N and C-O-C-, respectively.

**Fig. S2.** Cytocompatibility of native CO, CO-g-ChNCs_10_ and CO-g-ChNCs_10_/OCT_2.5_ after seeding with Saos-2 stem cells for one day, one week and three weeks (a, b). SEM of native CO hydrogel (c, d); CO-g-ChNCs_10_ (e, f) and CO-g-ChNCs_10_/OCT_2.5 (_g, h). Scale bars for (c, e, g) are 20 µm and for (d, f, h) are 10 µm. All samples were measured three times to calculate the standard division (n ± 3).

**Fig. S3.** SEM photos of the NHDF and Saos-2 spreading on the native CO, CO-g-ChNCs_10_ and CO-g-ChNCs_10_/OCT_2.5_ hydrogel after 24 h of culture. Scale bars was 100 µm.

**Fig. S4.** Piezoelectric constant as a function of the CO-V_f_ in CO-g-ChNCs hydrogel membrane (black squares) with exponential growth trend line (black dashed line). The extrapolated $d_{14}$ value for collagen$0 V/V$ (pristine ChNCs) was $0.036C/N$.
